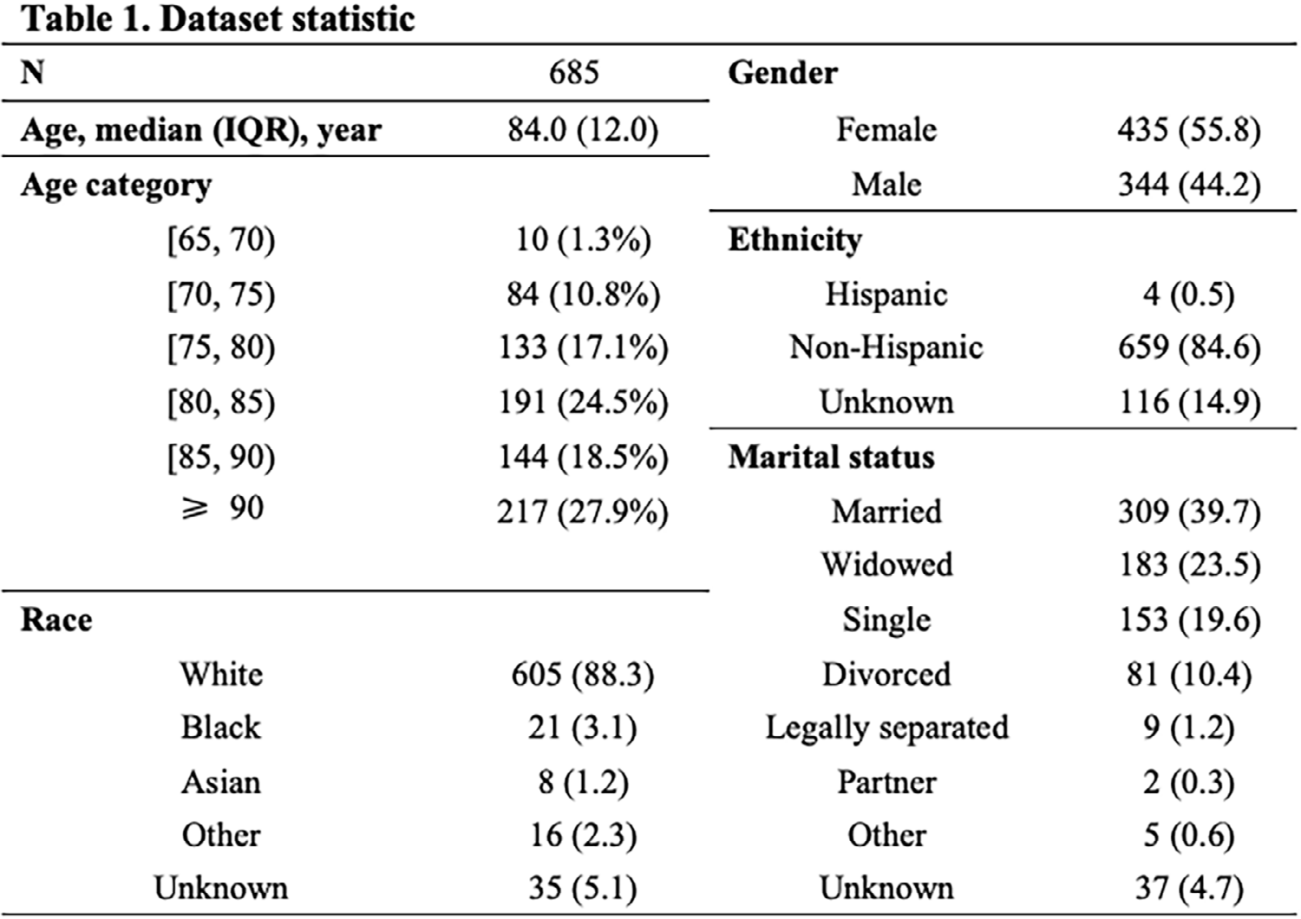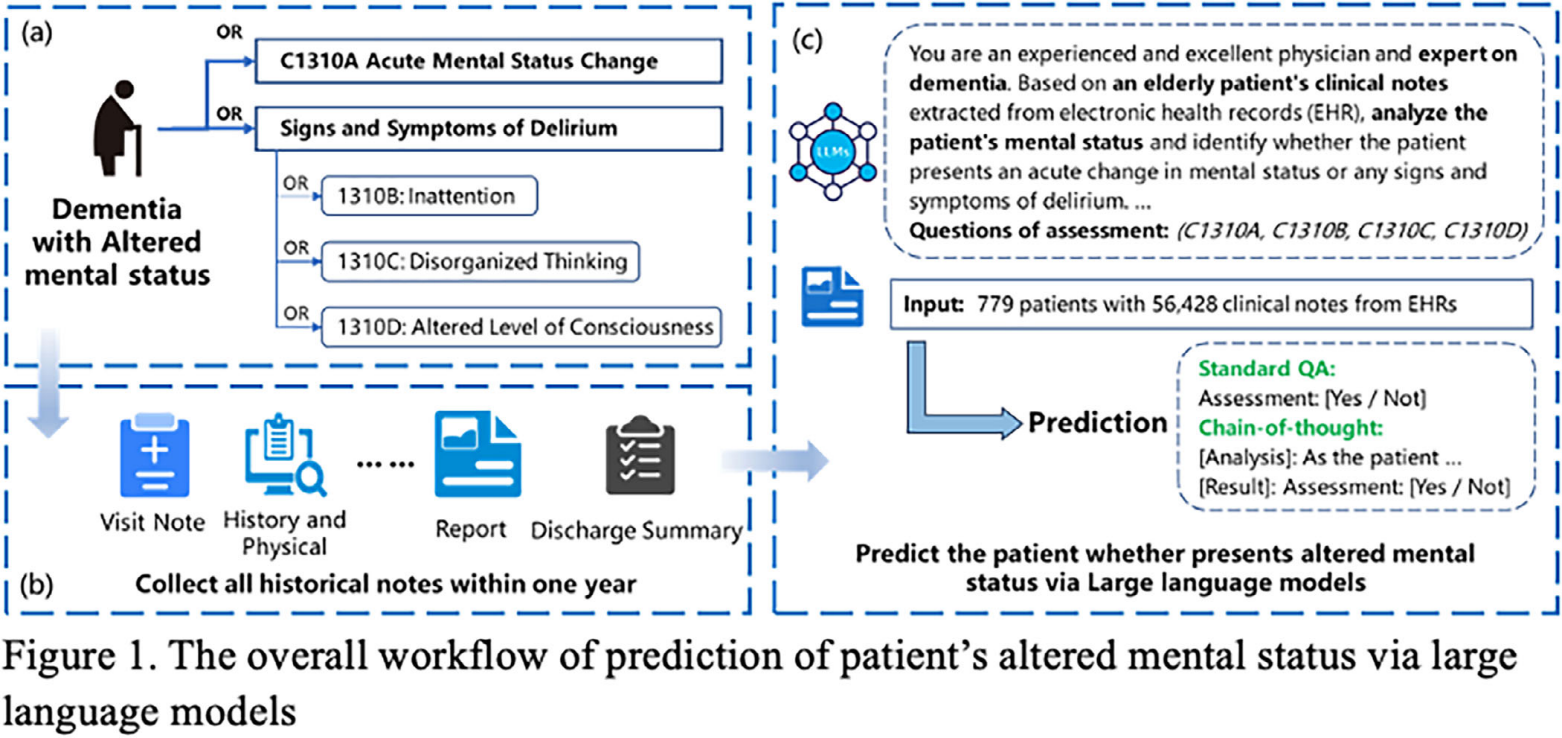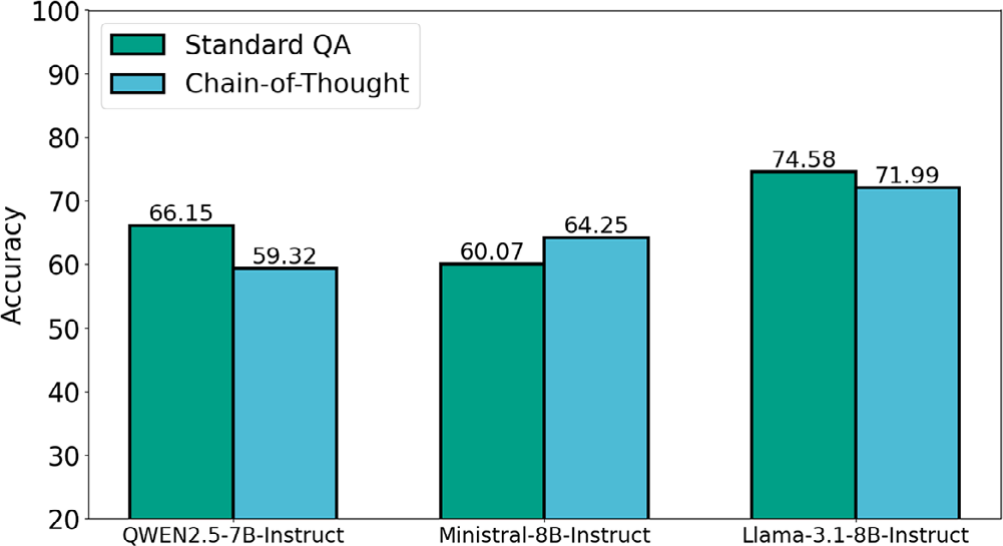# Large Language Model for Predicting Altered Mental Status in Dementia Patients Using Electronic Health Records

**DOI:** 10.1002/alz70858_104165

**Published:** 2025-12-26

**Authors:** Jiageng Wu, Richard Wyss, Josh Lin, Jie Yang

**Affiliations:** ^1^ Brigham and Women's Hospital, Boston, MA, USA

## Abstract

**Background:**

Older adults with dementia face substantial challenges to their mental status due to declining physical function and deteriorating lifestyle, which negatively impacts their quality of life. Mental status is a key determinant of daily functioning in progressive dementia, but assessments often require substantial resources and may be inaccessible to many. Leveraging Electronic Health Records (EHRs) and large language models (LLMs) to predict mental status offers a promising alternative for improving patient management, risk assessment, and timely interventions.

**Method:**

This retrospective cohort study analyzed EHRs of patients aged ≥65 years who were diagnosed with dementia between 2013 and 2020 and underwent Minimum Data Set (MDS) assessments at Mass General Brigham (MGB). Historical HER notes within the past year were utilized as model input. Altered mental status was defined based on the presence of C1310A Acute Mental Status Change or C1310 Signs and Symptoms of Delirium, including inattention, disorganized thinking, or altered level of consciousness. Three open‐source LLMs—QWEN2.5‐7B‐Instruct, Ministral‐8B‐Instruct, and Llama‐3.1‐8B‐Instruc were evaluated using two prompting strategies: a standard QA mode (direct answer output) and a Chain‐of‐Thought (CoT) mode (step‐by‐step reasoning). Performance was measured by accuracy using 1,000 bootstrapped iterations.

**Result:**

The study included 779 patients (median age: 84 years, 44.2% male) and their corresponding 56,428 clinical notes from the past year, extracted from EHRs. Among the models, Llama‐3.1‐8B‐Instruct achieved the highest accuracy of 74.58% (95% CI: [74.48, 74.68]) in the standard QA mode. The CoT strategy improved reasoning transparency and interpretability but led to a slight performance decline compared to the standard QA approach.

**Conclusion:**

This study demonstrates the potential of using EHRs and LLMs to predict altered mental status in dementia patients. The LLMs showed fine predictive performance, while the CoT strategy provided enhanced interpretability at the cost of reduced accuracy. These findings emphasize the need to optimize LLM in clinical applications and highlight the promise of advanced AI tools in improving dementia care through efficient risk assessment and timely interventions.